# Supplementary material for: Validation and reliability of the Bahasa Malaysia language version of the Acceptance of Illness Scale among Malaysian patients with cancer
Source: PLoS One. 2021 Sep 29;16(9):e0256216. doi: 10.1371/journal.pone.0256216 (PMC8480610; doi:10.1371/journal.pone.0256216)
Supplement: S1 Appendix — (PDF) [file pone.0256216.s001.pdf]

S1 APPENDIX

ACCEPTANCE OF ILLNESS SCALE (ENGLISH VERSION)

Instructions: Please circle the appropriate number to the right of each statement, indicating the level of acceptance of the disease. For an example, if the statement definitely applies to you, then you should circle the 1 in the first column. The participants evaluated statements on a scale from 1 (very poor acceptance to illness), 2 (poor acceptance to illness), 3 (average acceptance to illness), 4 (acceptance to illness), and 5 (fully acceptance to illness).

	**Definitely agree**	**Agree**	**Do not know**	**Do not agree**	**Definitely disagree**
1. I have problems with adjustment to the limitations imposed by the illness	1	2	3	4	5
2. Due to my state of health I am not able to do what I like best	1	2	3	4	5
3. The disease sometimes makes me feel unnecessary	1	2	3	4	5
4. Because of health problems I am more dependent on others than I wish to be	1	2	3	4	5
5. Due to the disease I am a burden on my family and friends	1	2	3	4	5
6. Due to my health status I do not feel a fully valued human being	1	2	3	4	5
7. I will never be self-sufficient to the degree I would like to be	1	2	3	4	5
8. I think that people who stay with me are often embarrassed because of my illness	1	2	3	4	5
