# Supplementary material for: Validation and reliability of the Bahasa Malaysia language version of the Acceptance of Illness Scale among Malaysian patients with cancer
Source: PLoS One. 2021 Sep 29;16(9):e0256216. doi: 10.1371/journal.pone.0256216 (PMC8480610; doi:10.1371/journal.pone.0256216)
Supplement: S2 Appendix — (PDF) [file pone.0256216.s002.pdf]

S2 APPENDIX

SKALA PENERIMAAN PENYAKIT (BAHASA MALAYSIA VERSION)

Arahan: Sila budarkan nombor yang sesuai di sebelah kanan setiap pernyataan, yang menunjukkan tahap penerimaan penyakit. Contohnya, jika pernyataan diberi berkaitan dengan anda, maka anda perlu bundarkan 1 dalam lajur pertama. Peserta menilai pernyataan dalam skala dari 1 (penerimaan sangat lemah terhadap penyakit), 2 (penerimaan buruk terhadap penyakit), 3 (penerimaan rata-rata terhadap penyakit), 4 (penerimaan terhadap penyakit), dan 5 (penerimaan sepenuhnya terhadap penyakit).

	**Sangat setuju**	**Setuju**	**Tidak tahu**	**Tidak setuju**	**Sangat tidak setuju**
1.Saya menghadapi masalah dengan penyesuaian terhadap batasan yang dikenakan oleh penyakit	1	2	3	4	5
2.Saya tidak dapat melakukan apa yang saya sukai dengan baik disebabkan oleh keadaan kesihatan saya	1	2	3	4	5
3.Penyakit ini kadang-kadang membuatkan saya berasa tidak diperlukan	1	2	3	4	5
4.Saya bergantung pada orang lain lebih daripada apa yang saya mahukan kerana masalah kesihatan	1	2	3	4	5
5.Saya menjadi beban kepada keluarga dan rakan-rakan saya oleh kerana penyakit ini	1	2	3	4	5
6.Saya tidak berasa seperti manusia yang dihargai sepenuhnya oleh kerana status kesihatan saya	1	2	3	4	5
7.Saya tidak dapat mencapai tahap yang saya inginkan	1	2	3	4	5
8.Saya merasakan bahawa orang yang tinggal bersama saya sering berasa malu disebabkan oleh penyakit saya	1	2	3	4	5
