# Supplementary material for: Validation and reliability of the Bahasa Malaysia language version of the Acceptance of Illness Scale among Malaysian patients with cancer
Source: PLoS One. 2021 Sep 29;16(9):e0256216. doi: 10.1371/journal.pone.0256216 (PMC8480610; doi:10.1371/journal.pone.0256216)
Supplement: Attachment — Submitted filename: Response to Reviewers (4.6).docx [file pone.0256216.s003.docx]

Review Comments to the Author:

Please use the space provided to explain your answers to the questions above. You may also include additional comments for the author, including concerns about dual publication, research ethics, or publication ethics. (Please upload your review as an attachment if it exceeds 20,000 characters)

Reviewer #1: It is very important to have research measures that are appropriate for different contexts and available in the language of choice of research participants. This study sought to validate a BM version of the Acceptance of Illness scale with a sample of cancer patients.

I think this manuscript would benefit from work to make the aims and objectives clearer. From the abstract it reads that this is the validation of an existing BM, but the main text makes it clear that the student actually did the translations. It would also benefit from some detailed proofing as in the PDF I received there were many cases of words mergedtogether.

In relation to the methods this study was carried out with recently diagnosed patients. I believe the original measure was developed using data from patients who were chronically ill. Could the authors explain why they chose the sample they did and how they took stage of illness into account?

**Response: We thank the reviewer for the comment. "Is it because at the beginning stage of cancer diagnosis, the illness is undoubtedly a source of stress, and may be perceived by the patient as a loss, threat, or challenge. These are also associated with illness acceptance, which begins as soon as the disease is suspected (Jankowska-Polanska et al., 2020). Patients may possible deal with illness acceptance challenges in accepting the reality, acceptance in treatment options, life routine adjustment, coping with irregular mood and emotions during their initial diagnosis period (Cipora et al., 2018). The effect on acceptance of illness has been evaluated from the initial of diagnosis, it is to help patients cope with their strategies, emotional management, illness acceptance and life adjustment in dealing with own illness (Kowalska et al., 2019). It was expected offering psychosocial support early as it could help cancer patients met with a high acceptance rate (Chan et al., 2015)."**

Inclusion and exclusion criteria should be outlined.

**Response: We thank the reviewer for the comment, we have now added the information on page 4, line 87-90 "The inclusion criteria were: 1) patients aged at least 18 years old and above; 2) able to understand and converse in Bahasa Malaysia; and 3) newly diagnosed with any cancer and first presentation at the oncology clinic. Patients who were too ill to tolerate an interview** **were excluded from the study."**

In relation to the test retest, I was unsure if this was a separate study or a sub study. It would be helpful to clarify this.

**Response: Our test-retest was done separately among 30 patients while 129 patients was recruited separately for validation. Thus, it is a separate analysis either.**

Construct validity is mentioned in the discussion, but no data is presented on this – I would recommend this be added.

**Response: We thank the reviewer for the comment and have added the following information." Table 2.Total variance explained in the component PCA."(page 10-11, line 173)**

Reviewer #2: This paper aims to translate the Acceptance of Illness Scale to the local language (Bahasa Malaysia), and validate and assess the reliability of the translated scale for use among cancer patients in Malaysia. The research are methodologically sound, with appropriate methods and statistical tests. The conclusion derived is supported by the findings of the study.

However this manuscript needs major revision before it can be published in the journal.

1. The English language is not up to standard, it makes reading difficult and affect understanding of what the authors are trying to say. It is strongly recommended that this manuscript is sent for English language proofreading.

**Response: We thank the reviewer for the comment, we will have sent to proofread.**

Methodology section:

2. Sample size of 129- how did the authors arrive at this number? Was there any sample size calculations done?

**Response: Yes we have done the calculation, the sample size calculation is based on the formulation of Peacock JL and Peacock PJ (2011) as follows:**

**n = [Z^2^ x (p) x(q)] / d^2^**

**Where:**

**n = sample size**

**Z = standard deviation of normal distribution (1.96 for 95% confidence interval)**

**d = confidence interval, which was set as 0.05**

**p = the proportion of the population with the desired characteristics. If the population size is unknown, it will be treated as a maximum variable and the p-value would be 0.50. In this study, the p-value was set at 0.10 as the prevalence rate of cancer at *Hospital Kuala Lumpur*, *Institute Kanser Negara* and *Pusat Perubatan Universiti Malaya* were estimated at 10%.**

**q =the proportion of the population without the desired characteristic, which can be calculated with formula 1-p.**

**Since the targeted population was fewer than 10,000 at the abovementioned locations of this study, sample adjustments ware done using the following formula:**

***nf* =	n**

**[(1+n) /N]**

**Where:**

***nf* = the desired sample size when the targeted population is fewer than 10,000**

**n = the calculated sample size**

**N = the total population**

**Therefore, the sample size needed for the study was:**

**N= [Z^2^ x (p) x(q)] / d^2^**

**N = [z^2^ x (p (1-p)] / d^2^**

**N = [1.96^2^ x (0.10 (1-0.10)] / 0.05^2^**

**N = [1.96^2^ x (0.10) x (0.90)] / 0.05^2^**

**N = 138 sample size needed**

**In total the study had recruited 150 patients however, there are 21 patients did not meet the inclusion criteria. Therefore, only 129 sample size can be used in the study.**

3. Need to explain who is Prof Tracey. Is she the owner/ developer of the scale?

**Response: We have now added that "the developer of the scale."(page 4, line 92)**

4. Need to clearly specify the inclusion and exclusion criteria

**Response: Thank you reviewer for pointing this out. We have clarify the inclusion and exclusion criteria in page 4, line 87-90"The inclusion criteria were: 1) patients aged at least 18 years old and above; 2) able to understand and converse in Bahasa Malaysia; and 3) newly diagnosed with any cancer and first presentation at the oncology clinic. Patients who were too ill to tolerate an interview** **were excluded from the study."**

5. Under instrument - Specify all the 5-degree scales ie 1 - poor adaptation to illness, 2 - ?, 3 - ?, 4 - ?, 5 - ?. Remove the 'for example 1 - definitely agree, 2 -agree........'

**Response: Thank you for reviewer for pointing this out. We have specific mention each of the scale "The acceptance of Illness Scale consists of eight items and is answered based on a five-point Likert scale, the participants evaluated statements on a scale from 1 (very poor acceptance to illness), 2 (poor acceptance to illness), 3 (average acceptance to illness), 4 (acceptance to illness), and 5 (fully acceptance to illness)."(page 5, line 104-107)**

6. Under Instrument- shouldn't the overall score of acceptance range between 5-40? Why did the author states it as 8-40?

**Response: Thank you for reviewer comment. "Based on the questionnaire consists of 8-items, and each of the item minimum score from 1 to maximum 5. When we sum up the total overall score of 8-items, minimum score is 8 to maximum 40. Thus, the range of total scores is from 8-40 which is from the total sum score of 8-items (Felton et al., 1984)".**

7. Under instrument - explain how cut-off points for high, average and low acceptance was obtained?

**Response: Thank you for reviewer comment. In page 5 (line 110-114)"In this study the cut-off points is based on the original tools designed by Felton et al. [20]. Participants were divided into three groups depending on the calculated Acceptance of Illness Scale score: (8–18 points) low acceptance of illness, (19–29 points) average acceptance of illness and (30–40 points) good acceptance of illness."**

8. Under translation - specify translation of scale to Bahasa Malaysia is by who and how many people

**Response: The following information has been added to offer additional context in page 5 (line 117-120). "The translation was carried out by three independent persons, comprising a psychologist, linguistic professional and a member of the general public, all of whom are proficient in both the English and Malay languages and had no prior knowledge of the scale."**

9. Under translation - remove the word 'about' 129 patients . You need to be very objective on the number of respondents included in the study

**Response: Thank you for pointing this out. We have removed the word 'about' in page 6 (line 143).**

Data analysis section:

10. Need to expand to include all the test that has been conducted ie pearson correlation coefficient etc

**Response: Thank you reviewer for comment. We have expanded and include all the related test which been conducted "Descriptive statistics were used to present participants' socio-demographic characteristics using frequencies, means, and standard deviations. To determine the validity of the Malay version of the Acceptance of Illness Scale, Principal Component Analysis (PCA) was used to examine the construct validity. The Bartlett’s test of sphericity was used to ascertain sampling adequacy, internal consistency, factors rotation and factors identification. Regarding extraction, the Kaiser's criterion and the scree plot were assessed. To evaluate the test-retest reliability, Pearson correlation coefficients was calculated by comparing the scores at the test and retest phases. Cronbach's alpha was calculated to assess the internal consistency of the scale."(Page 6, line 132-140)**

11. In the text mentioned chi square test, but the results of the test is not shown in results or discussed in discussion section.

**Response: Thank you for pointing this out, we have omitted specific mentioned of "Chi-squared was used to determine any variation across key variables", as this was based on another paper.**

Results section:
12. No need to mention about prospective study (n=150)

**Response: Thank you reviewer for pointing this out. We have remove this and rephrase the sentence to "A total of 129 cancer patients were recruited for this validation study." (Page 6, line 143-144)**

13. Is there a reason why the test-retest analysis is conducted on only 30 patients and not all 129?

**Response: Reason that we choose 30 patients for the test-retest it is based on Bujang and Baharum (2017) a guide to determine range of sample size requirements for test-retest was minimum from 10, therefore researchers would be recommended to collect an additional 20% to 30% to make up for any possible loss data due to drop-outs or missing data. A small sample size is usually required for estimation of level of consistency and this is preferable because test-retest reliability usually is conducted during an initial pilot study involving only a small sample size (Tan et al., 2013). In addition, it can be time consuming to perform a reliability estimation study by assessing the test-retest reliability.**

Discussion section:

14. KMO index is not part of the factor analysis test. It is done prior to conducting factor analysis to see the adequacy of dataset to conduct factor analysis. Please rephrase your sentence on KMO under discussion as well as abstract

**Response: We thank reviewer for the comment. We have rephrase our sentence on KMO under discussion and abstract "The Bartlett Test of Sphericity was statistical significance (χ²=366.321, p <0.001), while the Kaiser-Mayer-Olkin (KMO) measure of sampling adequacy was 0.84." (under abstract: Page 2, line 38-40); (under discussion: Page 10, line 162-164)**

15. Findings from Table 2 is not discussed at all under discussion section

**Response: We now have added information under discussion section. On the side note this has now edited to Table 3 "The mean score of the Acceptance of Illness in the sample was 29.01 (SD 6.74), demonstrating that the cancer patients in this study had moderate level of illness acceptance. Similar findings were reported by Budna et al. [23] who reported a mean score 28.30 among cancer patients after surgical treatment. On the other hand, Religioni et al. [24] reported a high level of illness acceptance with a mean score of 30.39 among cancer patients diagnosed with lung, breast, colorectal and prostate." (Page 16, line 236-241)**

16. There is a lot of redundancies/ repeated statements across the manuscript:
a) mentioned many many times that the scale consists of 8 items
b) mentioned few times that the scale can be used to measure level of acceptance of illness among cancer patients
under methodology
c) mentioned few times that scale is translated to Bahasa Malaysia under methodology

**Response: We thank reviewer for the comment. We now have acknowledged the repeated statements across the manuscript; we have made the needful correction.**

17. Repeated discussion on high internal consistencies with the same references quoted

**Response: We thank reviewer for pointing this out. We have made the needful amendment. "Internal consistency of the Malay version of the scale was found to be high in the current study. This is similar to that of the English version [20], [25-27] among patients with chronic illness (namely hypertension, diabetes, arthritis cancer). In this study, the Cronbach alpha value did not change excessively when the items were deleted. Thus, the current study showed that the translated scale had sufficient homogeneity with good reliability." (Page 17, line 253-258)**

18. Please check the formatting of tables

**Response: We have check and amended the formatting tables based on Plos one requirement.**

Reviewer #3:

1) This is an important manuscript for South East Asians as Bahasa Malaysia is the main language used in Malaysia and in addition, it may also be used in Indonesia as well which has a large population that uses Bahasa Melayu.

2) In the last paragraph of the Introduction, it would be useful for the author to describe the multi-ethnic and multi-lingual society of Malaysia, including the estimated users of Bahasa Malaysia as the national language and to highlight the necessity to have this scale translated and validated for the local population where English is not the main language used.

**Response: We have modified the following paragraph in page 4 (line 76-82) to address the valid points raised by the reviewer: "Malaysia consists of a multi-ethnic population [14] that comprised Malay, Chinese, Indians, and others (Iban, Kadazan, orang asli) [15]. The national language of Malaysia is the Malay language (*Bahasa Malaysia*). We aimed to translate and validate a Bahasa Malaysia version of the Acceptance of Illness scale, and to determine the psychometric properties of the translated scale. The translated instrument can be used to evaluate the level of illness acceptance in the oncology settingamong the local Malay-speaking population of cancer patients."**

3) In terms of the descriptive analysis, the cohort in this study has 56.3% with secondary school education, 14.1% with university and 16.4 with college / diploma education, which seems to be quite a highly educated cohort. Is this comparable to the Malaysia population generally? Please comment either way.

**Response: Thank you for reviewer comment. "Based on Malaysia's population 2020 estimated at 32.7 million as compared to 32.5 million in 2019 with an annual growth rate of 0.4 per cent (Department of Statistics Malaysia, 2020).According to Sani et al., 2020 study, in 2018, it was estimated that 4.1 million households belong to B40 group which classify in bottom income (not more than RM 4,630.00). The government of Malaysia has widened access to higher education for the B40 group in an effort to reduce the gaps in socioeconomics and to improve their living standards. As reported in Higher Education Statistic, from the year 2011 to 2015, the total number of students intake for bachelor's degree programmes in Malaysia Public Universities is more than 85,000 students yearly. The highest number of students intake was recorded in the year 2011 with 99,862 students. Higher education institution and skills training institutes were encouraged to allocate more seats and allowing admission by special allocation for B40 students in an effort to ensure their access to higher education is secured. In terms of post-secondary education in 2016, 20,232 students were enrolled in public community colleges, 99,551 students in public polytechnics, 695,026 in private higher education institutions and 532,049 across the twenty public universities in Malaysia (Ministry of Higher Education, 2017)."**

4) In the discussion on page 14, this study had a 2 week test-retest reliability score which is a shorter period compared to other studies. Can the author comment on this as oncology newly diagnosed patients are usually seen within a much longer period to complete their treatment.

## Response: Thank you for reviewer comment. "Based on Blick et al. (2010) mentioned that 2 week wait rule on patient with newly diagnosis and treatment of bladder cancer has reduced the time for the overall pathway as well as the time taken to be seen by a specialist. In Malaysia oncology centres practices, newly diagnosed patients appointment will be given in 2 weeks time to review specialist for treatment decision and treatment plan. Patients then will be retest on their second appointment (2 weeks), while they were on the waiting to see specialist for treatment decision and treatment plan follow up. This is to ensure that the patients have feelings of preparedness in dealing with cancer, and coping prior treatment commence."
