# Supplementary material for: Validation and reliability of the Bahasa Malaysia language version of the Acceptance of Illness Scale among Malaysian patients with cancer
Source: PLoS One. 2021 Sep 29;16(9):e0256216. doi: 10.1371/journal.pone.0256216 (PMC8480610; doi:10.1371/journal.pone.0256216)
Supplement: Attachment — Submitted filename: Response to Reviewer (13.7).docx [file pone.0256216.s004.docx]

## 6. Review Comments to the Author

## Please use the space provided to explain your answers to the questions above. You may also include additional comments for the author, including concerns about dual publication, research ethics, or publication ethics. (Please upload your review as an attachment if it exceeds 20,000 characters)

## Reviewer #3: Thank you for your revision. For comment no 4, your response was

## "In Malaysia oncology centres practices, newly diagnosed patients appointment will be given in 2 weeks time to review specialist for treatment decision and treatment plan. Patients then will be retest on their second appointment (2 weeks), while they were on the waiting to see specialist for treatment decision and treatment plan follow up. This is to ensure that the patients have feelings of preparedness in dealing with cancer and coping prior treatment commence." Please summarise this paragraph and add on to the discussion of the paper to highlight the reason why the re-test is 2 weeks and not longer.

Response: **We now add on the reasons why the test-retest is in 2 weeks and not longer in page 15 (line 274-279) "We conducted the test-retest reliability after a two week period, which is a shorter period compared to other studies, because in Malaysia oncology centres practice the two weeks’ wait rule with newly diagnosis patients for their oncologist to review their treatment discussion and decision follow-up. This is to ensure patients have better self-emotion adjustment and mental preparedness before the start of treatment."**
